# Simultaneous control of the mechanical properties and adhesion of human umbilical vein endothelial cells to suppress platelet adhesion on a supramolecular substrate†

**DOI:** 10.1039/d2ra04885j

**Published:** 2022-09-30

**Authors:** Junsu Park, Tomoya Ueda, Yusaku Kawai, Kumiko Araki, Makiko Kido, Bunsho Kure, Naomi Takenaka, Yoshinori Takashima, Masaru Tanaka

**Affiliations:** Department of Macromolecular Science, Graduate School of Science, Osaka University 1-1 Machikaneyama Toyonaka Osaka 560-0043 Japan; Institute for Advanced Co-Creation Studies, Osaka University 1-1 Yamadaoka Suita Osaka 565-0871 Japan takasima@chem.sci.osaka-u.ac.jp; Institute for Materials Chemistry and Engineering, Kyushu University CE41 744 Motooka, Nishi Fukuoka 819-0395 Japan masaru_tanaka@ms.ifoc.kyushu-u.ac.jp; Forefront Research Center, Graduate School of Science, Osaka University 1-1 Machikaneyama Toyonaka Osaka 560-0043 Japan; Innovative Catalysis Science Division, Institute for Open and Transdisciplinary Research Initiatives (OTRI), Osaka University 1-1 Yamadaoka Suita Osaka 565-0871 Japan; Nara Laboratory, Kyoeisha Chemical Co., Ltd 2-5,5-chome, Saikujo-cho Nara 630-8453 Japan

## Abstract

The demand for artificial blood vessels to treat vascular disease will continue to increase in the future. To expand the application of blood-compatible poly(2-methoxyethyl acrylate) (pMEA) to artificial blood vessels, control of the mechanical properties of pMEA is established using supramolecular cross-links based on inclusion complexation of acetylated cyclodextrin. The mechanical properties, such as Young's modulus and toughness, of these pMEA-based elastomers change with the amount of cross-links, maintaining tissue-like behavior (J-shaped stress–strain curve). Regardless of the cross-links, the pMEA-based elastomers exhibit low platelet adhesion properties (approximately 3% platelet adherence) compared with those of poly(ethylene terephthalate), which is one of the commercialized materials for artificial blood vessels. Contact angle measurements imply a shift of supramolecular cross-links in response to the surrounding environment. When immersed in water, hydrophobic supramolecular cross-links are buried within the interior of the materials, thereby exposing pMEA chains to the aqueous environment; this is why supramolecular cross-links do not affect the platelet adhesion properties. In addition, the elastomers exhibit stable adhesion to human umbilical vein endothelial cells. This report shows the potential of combining supramolecular cross-links and pMEA.

## Abstract 2

Supramolecular cross-links in poly(2-methoxyethyl acrylate) enhanced mechanical properties of the polymers maintaining high blood compatibility. The high blood compatibility suggests a potential for artificial blood vessel.

## Graphical abstract

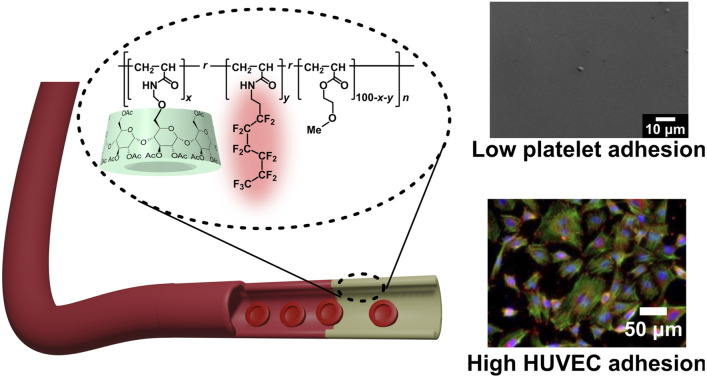

## Introduction

As one treatment for ischemic heart disease, which is a leading cause of death, replacement of the diseased vessel with an artificial vessel can be considered. Several companies have commercialized artificial vessels based on, for example, poly(ethylene terephthalate) (PET) and poly(tetrafluoroethylene).^1,2^ Although these polymers have been commercially implemented in biocompatible devices, surface thrombogenicity has limited the application of these materials as artificial vessels.^3–5^ To overcome these issues, several approaches have been studied, such as surface modification, immobilization of blood compatible cells, and the search for new materials.^6^ Regarding surface modification, various polymers such as poly(ethylene glycol),^7–10^ poly(vinyl pyrrolidone),^11,12^ sulfobetaine-based polymers,^13–15^ and zwitterionic polyurethane^16^ have been investigated. These modified polymers utilized repulsion forces between the protein in blood and the modified surface.

For artificial blood vessels, scientists have sought polymeric materials such as silk,^17–21^ collagen,^22,23^ and chitosan^24–26^ as natural polymers^27,28^ and poly(2-methoxyethyl acrylate) (pMEA),^29,30^ poly(2-methacryloyloxyethyl phosphorylcholine),^31–33^ and polycaprolactone^34–37^ as synthetic polymers. Among the synthetic polymers, pMEA and pMEA-like polymers showed outstanding blood compatibility, with simultaneously low platelet adhesion and high human umbilical vein endothelial cell (HUVEC) adhesion.^38^ Later, HUVEC-coated pMEA was recognized as native tissue for blood. Although pMEA and pMEA-like polymers^39^ are biocompatible, they are only applicable as coatings due to their low mechanical properties (in particular, lack of elasticity).

**Fig. 1:**
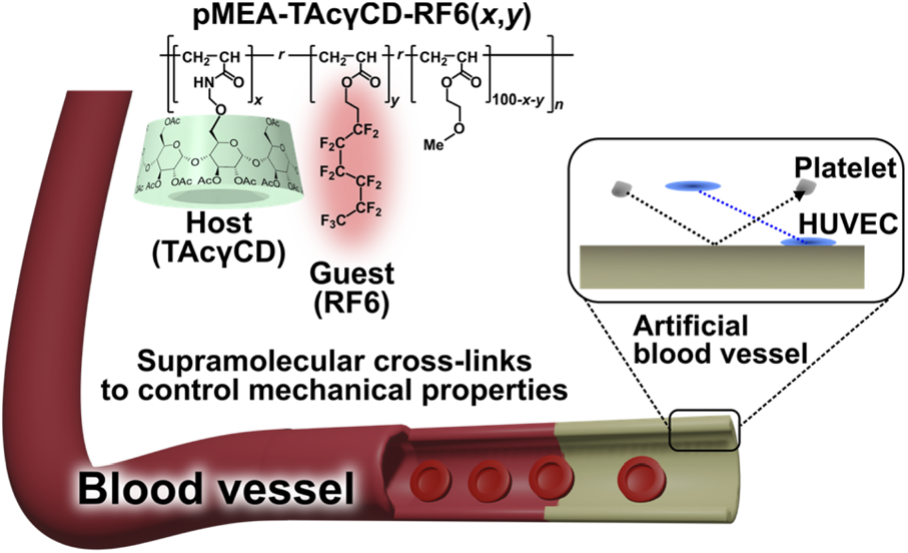
Chemical structure and conceptual illustration of a poly(2-methoxyethyl acrylate) (pMEA)-based polymeric materials (pMEA–TAcγCD–RF6 (*x*,*y*), where *x* and *y* refer to the mol% of TAcγCD and RF6, respectively) for artificial blood vessels. Platelets must not adhere to the artificial blood vessel, while human umbilical vein endothelial cells (HUVECs) should adhere. Ultimately, the vessel should be coated by HUVECs to be recognized as a biomaterial for blood to prevent the formation of a thrombus.

Herein, we established a methodology to control the mechanical properties of pMEA-based polymeric materials and studied their blood compatibility to evaluate the potential for artificial blood vessels (Fig. 1). We incorporated supramolecular cross-links consisting of acetylated γ-cyclodextrin (TAcγCD) and a perfluorohexane-based guest (RF6) to control the mechanical properties. These supramolecular cross-links based on host–guest chemistry have been shown to significantly improve the mechanical properties of polymeric materials.^40–44^ First, we prepared pMEA-based elastomers with supramolecular cross-links and characterized them with nuclear magnetic resonance (NMR) measurements and Fourier transform infrared (FT-IR) spectroscopy. Subsequently, the control of mechanical properties was investigated by tensile tests. Blood compatibility was confirmed by observation of adhered platelets and HUVECs on the polymeric materials.

## Results and discussion

### Preparation of elastomers

We prepared pMEA-based elastomers with TAcγCD (host) and RF6 (guest) in various ratios (Schemes S1, S2, Tables S1 and S2†). For each tensile test and cell adhesion test, LED lamp and Hg lamp were used as the light sources respectively, as the LED lamp was not appropriate to prepare large amounts of elastomers for the cell adhesion tests. The obtained polymers were named pMEA–TAcγCD–RF6 (*x*,*y*), where *x* and *y* refer to the mol% of TAcγCD and RF6 in the feeding ratio, respectively. As representative elastomers prepared with the Hg lamp, four different types, namely, pMEA–TAcγCD–RF6 (0,0), pMEA–TAcγCD–RF6 (1,0), pMEA–TAcγCD–RF6 (0,1) and pMEA–TAcγCD–RF6 (1,1), were analyzed by ^1^H NMR measurements (Fig. S1–S4†). The peaks of pMEA and TAcγCD in the ^1^H NMR spectra implied that the desired polymers were obtained when (*x*,*y*) = (0,0) and (1,0). However, it was difficult to reliably confirm the existence of RF6 units in pMEA–TAcγCD–RF6 (0,1) and pMEA–TAcγCD–RF6 (1,1) by ^1^H NMR measurements.

**Fig. 2:**
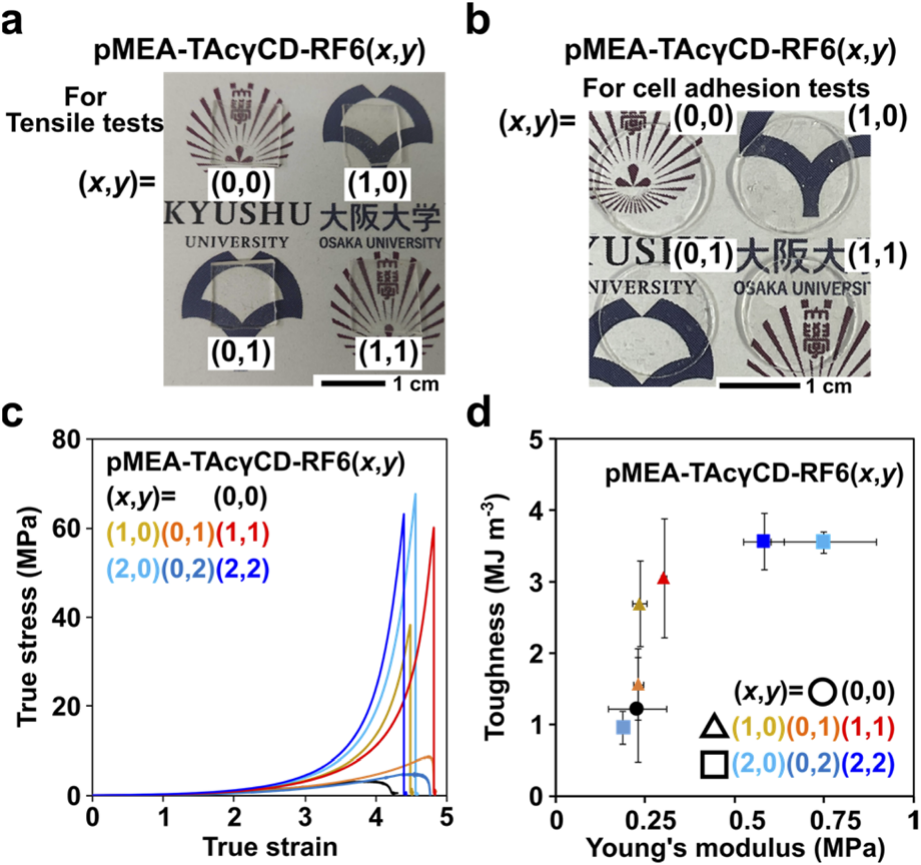
(a) Photographs of pMEA–TAcγCD–RF6 (0,0), (1,0), (0,1) and (1,1) for tensile tests. (b) Photographs of pMEA–TAcγCD–RF6 (0,0), (1,0), (0,1) and (1,1) cut into disk shapes for cell adhesion tests. (c) Stress–strain curve of pMEA–TAcγCD–RF6 (*x*,*y*) expressed in true stress and true strain. (d) Two-dimensional plot of toughness *versus* Young's modulus of pMEA–TAcγCD–RF6 (*x*,*y*). Error bars refer to standard deviations.

^19^F NMR measurements were additionally performed to confirm the presence of RF6 because of the simple detection of fluorine-based compounds. For the ^19^F NMR measurements of the elastomers, a trifluoroacetic acid (TFA) solution in CDCl_3_ was measured to optimize the shim and lock settings prior to assessing the four elastomers instead of direct addition as an internal standard, since TFA can promote the deprotection of acetyl groups on TAcγCD and cleavage of the hemiaminal group connecting TAcγCD and pMEA (Fig. S5†). Subsequently, ^19^F NMR measurements of the four elastomers were carried out using the appropriate settings (Fig. S6–S9†). The ^19^F NMR spectra of pMEA–TAcγCD–RF6 (0,0) and pMEA–TAcγCD–RF6 (1,0), which contain no fluorine-based units, showed no visible signals. In contrast, the ^19^F NMR spectra of pMEA–TAcγCD–RF6 (0,1) and pMEA–TAcγCD–RF6 (1,1) had six peaks in the range of −125 to −75 ppm, suggesting the existence of fluorine-based units. According to the ^1^H and ^19^F NMR measurements, we concluded that the desired polymers were obtained by Scheme S1.† The FT-IR spectroscopy results also supported the polymeric structures (Fig. S10†). The four obtained pMEA–TAcγCD–RF6 (*x*,*y*) elastomers were transparent regardless of the light sources (Fig. 2a and b).

### Mechanical properties of the elastomers

Mechanical properties are important parameters for artificial blood vessels. We investigated the mechanical properties of pMEA–TAcγCD–RF6 (*x*,*y*) by tensile tests (Fig. 2c). According to a previous report, the shape of the stress–strain curve expressed in true stress is a characteristic feature of materials.^45^ It was reported that tissue has a J-shaped stress–strain curve to achieve simultaneous softness (low Young's modulus) and difficulty to fracture. pMEA–TAcγCD–RF6 (*x*,*y*) elastomers with TAcγCD alone or TAcγCD and RF6 showed J-shaped stress–strain curves, regardless of the TAcγCD and RF6 ratio, suggesting potential application in artificial blood vessels.

The toughness and Young's modulus of pMEA–TAcγCD–RF6 (*x*,*y*) were calculated from the area between the stress–strain curves expressed in engineering stress and strain and strain axes and the initial slope of the curves, respectively (Fig. S11†). Toughness and Young's moduli were plotted in a two-dimensional plot for comprehensive comparison of the mechanical properties (Fig. 2d). The toughness of the materials was mainly dominated by ultimate strength defined as engineering stress at fracture point. Polymeric materials without cross-links ((*x*,*y*) = (0,0), (0,1), and (0,2)) had the lowest toughness than the other materials. When (*x*,*y*) = (1,0), (0,1) and (1,1), their Young's moduli were similar to that of pMEA ((*x*,*y*) = (0,0)). In contrast, with (*x*,*y*) = (2,2), the polymeric materials showed the largest Young's modulus, followed by (2,0) and (0,2). These two results indicate that incorporating supramolecular cross-links into pMEA-based elastomers allows control of the mechanical properties.

### Platelet adhesion properties on the elastomers

**Fig. 3:**
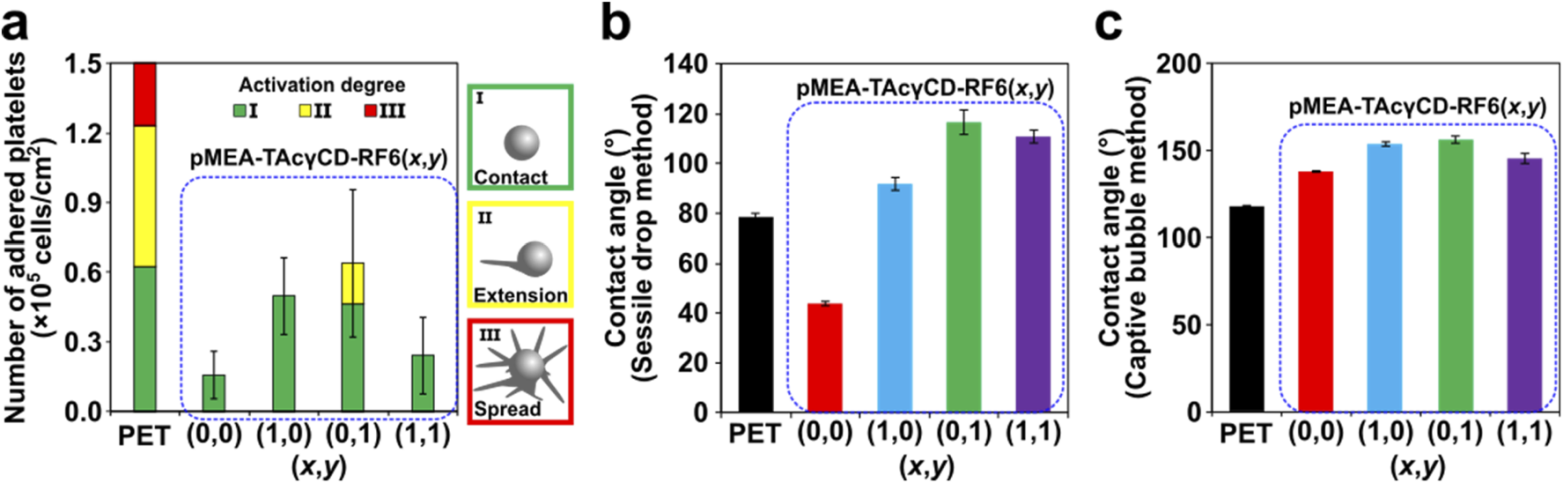
(a) The numbers and activation degrees of adhered platelets on pMEA–TAcγCD–RF6 (*x*,*y*) and PET as a reference material. (b) Contact angles of pMEA–TAcγCD–RF6 (*x*,*y*) and PET by the sessile drop method 30 seconds after dropping. (c) Contact angles of pMEA–TAcγCD–RF6 (*x*,*y*) and PET by the captive bubble method one hour after forming an air bubble.

Young's moduli of blood vessels are expected to range from 0.2–0.6 MPa.^46^ Based on this value range, we carried out cell adhesion tests focusing on pMEA–TAcγCD–RF6 (*x*,*y*) when (*x*,*y*) = (0,0), (1,0), (0,1) and (1,1). A platelet suspension (seeding density = 4 × 10^7^ cells per cm^2^) was dropped and incubated on the four pMEA-based materials and PET as a reference material (Scheme S3†). Subsequently, we observed the surface of the materials by scanning electron microscopy (SEM) (Fig. S12†). The PET surface showed significantly adhered platelets. Some of the platelets presented a deformed morphology on the substrates. On the other hand, pMEA–TAcγCD–RF6 (*x*,*y*) showed almost no platelet adhesion, compared with the surface before the cell adhesion tests. We defined the activation degrees of the platelets as three steps according to previous reports: (I) primary contact, (II) extension of filopodia, and (III) fully spread platelets.^47,48^ Subsequently, we counted the number of adhered platelets with their activation degrees (Fig. 3a and S13†). It was revealed that the pMEA-based materials suppressed adhesion of the platelets regardless of *x* and *y*, maintaining a relatively lower activation degree. Among the four pMEA-based materials, pMEA–TAcγCD–RF6 (0,0) and pMEA–TAcγCD–RF6 (1,1) showed the lowest numbers of adhered platelets.

To understand the results, we investigated the contact angles of the polymeric materials. First, we measured the contact angles by the sessile drop method (Fig. 3b). The contact angle on PET was approximately 80°, implying a relatively hydrophobic surface compared with that of pMEA–TAcγCD–RF6 (0,0), whose contact angle was approximately 40°. Adding TAcγCD or RF6 to pMEA drastically increased the hydrophobicity of the materials. The high hydrophobicity was attributed to acetyl groups of TAcγCD and the fluorinated chain of RF6. The contact angle determined by the sessile drop method corresponds to the sum of the amounts of intermediate water and non-freezing water.^49^ More intermediate water and non-freezing water result in a lower contact angle. Accordingly, the hydrated water, in particular intermediate water, is considered the reason for the blood compatibility of pMEA–TAcγCD–RF6 (0,0).^38^

However, it was difficult to discuss the adhesion properties of platelets for pMEA–TAcγCD–RF6 (*x*,*y*), except for (*x*,*y*) = (0,0), using the contact angles obtained by the sessile drop method. We used the captive bubble method for further studies on the surface properties (Fig. 3c). PET and pMEA–TAcγCD–RF6 (0,0) maintained their hydrophobicity or hydrophilicity regardless of the surrounding environment and were relatively hydrophobic and hydrophilic, respectively. Interestingly, other pMEA–TAcγCD–RF6 (*x*,*y*) elastomers became hydrophilic when surrounded by water (hydrated). The contact angles of pMEA–TAcγCD–RF6 (1,0), pMEA–TAcγCD–RF6 (0,1) and pMEA–TAcγCD–RF6 (1,1) obtained by the captive bubble method were similar to or higher than that of pMEA–TAcγCD–RF6 (0,0). The drastic changes in the surface states can be explained by structural reorganization of the surface as suggested in previous reports.^50–52^ Under air, hydrophobic TAcγCD and RF6 are located at the surface close to air, resulting in a hydrophobic surface. In contrast, when hydrated, hydrophilic pMEA polymeric chains appear at the surface, with the hydrophobic moieties buried within the interior of the materials. Consequently, all pMEA–TAcγCD–RF6 (*x*,*y*) materials behave like pMEA–TAcγCD–RF6 (0,0) and inhibit the adhesion of platelets due to the hydrated pMEA polymeric chains at the surface.

### HUVEC adhesion properties on the elastomers

Because pMEA–TAcγCD–RF6 (*x*,*y*) showed potential as an alternative to pMEA–TAcγCD–RF6 (0,0), we investigated the HUVEC adhesion properties on pMEA–TAcγCD–RF6 (*x*,*y*) as a substrate. The surfaces of all samples were washed with phosphate-buffered saline (PBS). Subsequently, HUVECs at a specific density (1.0 × 10^4^ cells per cm^2^) were seeded on the samples. After 1, 24, and 72 hours-incubation, the adhered cells were fixed, permeabilized and stained. The numbers and morphologies of the adhered cells were mainly studied (Table S3†). After 1 hour of incubation, the numbers of adhered HUVECs were similar, although that of pMEA–TAcγCD–RF6 (0,0) was 2–30% larger than the other values. The morphology observed by fluorescence microscopy was different for the polymers (Fig. S14†). The HUVECs on PET and pMEA–TAcγCD–RF6 (1,1) were relatively round, while those on the other substrates were extended. The numbers of adhered HUVECs increased after 24 hours of incubation. In particular, PET resulted in a drastic increase in the number of adhered HUVECs, while the number of HUVECs on the other substrates only slightly increased, implying a stabilized state.

**Fig. 4:**
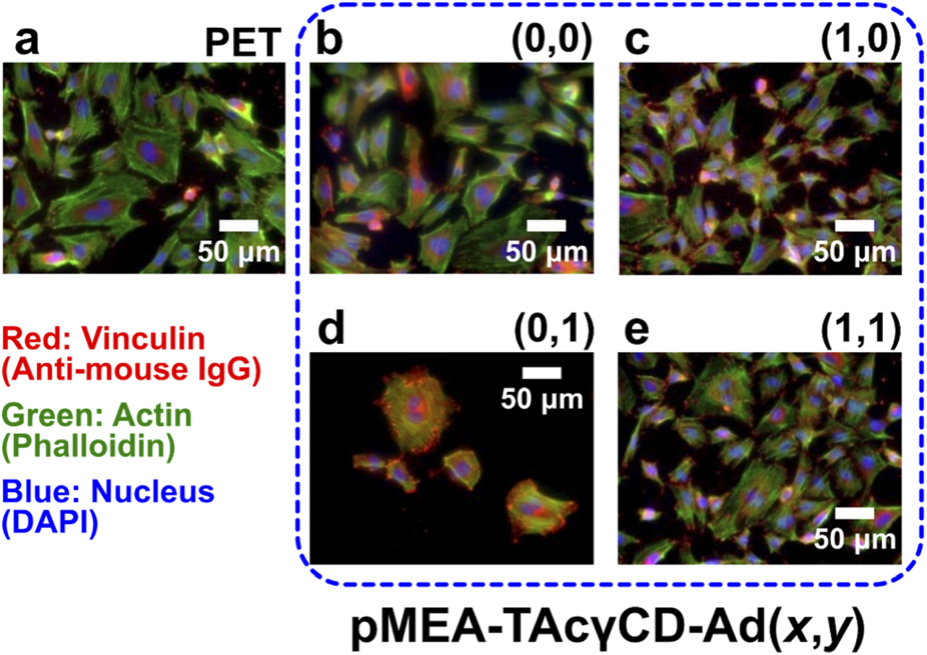
Fluorescence microscopic images of adhered HUVECs after 24 hours of incubation on (a) PET, pMEA–TAcγCD–RF6 (b) (0,0), (c) (1,0), (d) (0,1), and (e) (1,1). For better comparison, the contrast and brightness of the images were controlled. Vinculin, actin, and nuclei were stained with anti-mouse IgG, phalloidin, and 4′,6-diamidino-2-phenylindole (DAPI), respectively.

As 24 hours of incubation seemed enough to equilibrate the incubation, we quantitatively evaluated the morphologies of the adhered HUVECs based on four standards, namely, area, circularity, aspect ratio, and roundness (Table S4†), as well as microscopic images (Fig. 4). In terms of the area of the adhered HUVECs, PET, pMEA–TAcγCD–RF6 (1,0) and pMEA–TAcγCD–RF6 (0,1) resulted in an approximately 35% larger area, an approximately 32% smaller area, and an unusually larger area (approximately 370% of that on pMEA–TAcγCD–RF6 (0,0)), respectively, while the other materials resulted in similar cell areas. The circularities of the adhered HUVECs were similar (less than 10% error) regardless of the substrate. The aspect ratios of the HUVECs were similar (∼2), except for those on pMEA–TAcγCD–RF6 (0,1), on which the aspect ratio was approximately 30% smaller than that on pMEA–TAcγCD–RF6 (0,0). Regarding roundness, only pMEA–TAcγCD–RF6 (0,1) resulted in a 37% larger value. The adhered HUVECs showed unusual morphologies on PET, pMEA–TAcγCD–RF6 (1,0), and pMEA–TAcγCD–RF6 (0,1); therefore, pMEA–TAcγCD–RF6 (1,1) appeared to be the most appropriate candidate for artificial blood vessels coated with HUVECs. Furthermore, the numbers of adhered HUVECs on pMEA–TAcγCD–RF6 (1,0) and pMEA–TAcγCD–RF6 (0,1) drastically decreased after 72 hours of incubation, while pMEA–TAcγCD–RF6 (1,1) showed stable HUVEC adhesion. In addition, we carried out the HUVEC adhesion test on a tissue culture polystyrene (TCPS) dish as a positive control (Fig. S15†). The morphologies of the adhered HUVECs on TCPS were similar to those on pMEA–TAcγCD–RF6 (0,0) and pMEA–TAcγCD–RF6 (1,1). These results also support the potential of pMEA–TAcγCD–RF6 (1,1) as an artificial blood vessel material.

## Conclusions

In this study, we prepared mechanical property-controllable blood-compatible pMEA utilizing supramolecular cross-links between TAcγCD and a perfluorohexane-based guest (RF6). pMEA elastomers with supramolecular cross-links were simply obtained by free radical copolymerization of 2-methoxyethyl acrylate with TAcγCD and perfluorohexane-based monomers. The cross-link content affected the mechanical properties of the pMEA-based elastomers, maintaining tissue-like tensile behavior (J-shaped curves on the true stress *versus* true strain curve). When the materials contained 1 mol% TAcγCD and/or RF6, their Young's moduli were similar to those of blood vessels. SEM analysis showed that the pMEA-based elastomers exhibited low platelet adhesion properties similar to those of pMEA, regardless of the contents of TAcγCD and RF6. According to contact angle tests, the similar hydrophilicity of pMEA-based elastomers to that of pMEA seems to be the reason why all pMEA-based elastomers showed low platelet adhesion properties. Moreover, HUVECs adhered stably to pMEA-based elastomers containing 1 mol% TAcγCD and RF6 without significant morphological changes, implying the potential as an artificial blood vessel material.

Artificial blood vessels will be required in the future to help treat vascular diseases. Blood-compatible pMEA is usually applicable as a coating for medical apparatuses such as stents because it is impossible to control mechanical properties. The present report suggests an effective approach to control the mechanical properties of pMEA based on supramolecular chemistry. Eventually, pMEA-based elastomers could be applicable in various manners for artificial blood vessels.

## Author contributions

J. P. and T. U. equally contributed to this work. J. P.: investigation, methodology, and writing – original draft, T. U.: investigation and writing – original draft, Y. K.: investigation, K. A.: investigation, M. K.: investigation, B. K.: resource, funding acquisition, N. T.: resource, funding acquisition, Y. T.: conceptualization, funding acquisition, and writing – review and editing. M. T.: conceptualization, funding acquisition, and writing – review and editing.

## Conflicts of interest

There are no conflicts to declare.

## Supplementary Material
